# Soluble receptors for advanced glycation end-products prevent unilateral ureteral obstruction-induced renal fibrosis

**DOI:** 10.3389/fphar.2023.1172269

**Published:** 2023-05-16

**Authors:** Chan Ho Kim, Hye-Young Kang, Gyuri Kim, Jimin Park, Bo Young Nam, Jung Tak Park, Seung Hyeok Han, Shin-Wook Kang, Tae-Hyun Yoo

**Affiliations:** ^1^ Department of Internal Medicine, International St. Mary’s Hospital, Catholic Kwandong University College of Medicine, Incheon, Republic of Korea; ^2^ Department of Internal Medicine, College of Medicine, Severance Biomedical Science Institute, Brain Korea 21 Project for Medical Science, Yonsei University, Seoul, Republic of Korea; ^3^ Department of Internal Medicine, College of Medicine, Severance Biomedical Science Institute, Institute of Kidney Disease Research, Yonsei University, Seoul, Republic of Korea

**Keywords:** receptor for advanced glycation end-products (RAGE), soluble RAGE, unilateral ureteral obstruction, renal fibrosis, chronic kidney disease

## Abstract

**Introduction:** The receptor for advanced glycation end products (RAGE) and its ligands, such as high-mobility group protein box 1 (HMGB1), play an important role in the accumulation of extracellular matrix in chronic kidney diseases with tubulointerstitial fibrosis. Blocking RAGE signaling with soluble RAGE (sRAGE) is a therapeutic candidate for renal fibrosis.

**Methods:** NRK-52E cells were stimulated with or without HMGB1 and incubated with sRAGE *in vitro*. Sprague-Dawley rats were intraperitoneally treated with sRAGE after unilateral ureteral obstruction (UUO) operation *in vivo*.

**Results:** HMBG1-stimulated NRK-52E cells showed increased fibronectin expression, type I collagen, *α*-smooth muscle actin, and connective tissue growth factor, which were attenuated by sRAGE. The mitogen-activated protein kinase (MAPK) pathway and nuclear translocation of nuclear factor kappa B (NF-κB) were enhanced in NRK-52E cells exposed to HMBG1, and sRAGE treatment alleviated the activation of the MAPK and NF-κB pathways. In the UUO rat models, sRAGE significantly ameliorated the increased renal fibronectin, type I collagen, and α-smooth muscle actin expressions. Masson’s trichrome staining confirmed the anti-fibrotic effect of sRAGE in the UUO rat model. RAGE also significantly attenuated the activation of the MAPK pathway and NF-κB, as well as the increased number of infiltrated macrophages within the tubulointerstitium in the kidney of the UUO rat models.

**Conclusion:** These findings suggest that RAGE plays a pivotal role in the pathogenesis of renal fibrosis and that its inhibition by sRAGE may be a potential therapeutic approach for renal fibrosis.

## 1 Introduction

Tubulointerstitial fibrosis, characterized by the accumulation of fibroblasts and extracellular matrix (ECM) within the interstitium of the kidney along with the loss of functioning nephrons, is a major pathological feature of progressive chronic kidney disease (CKD). (Boor et al., 2010; Liu, 2011) Moreover, interstitial inflammation and fibrosis within the kidney are significant risk factors for the progression of CKD toward end-stage renal disease. (Becker and Hewitson, 2000; Verma et al., 2021; Ravarotto et al., 2022) despite many therapeutic interventions having been tried, an effective therapy to mitigate renal fibrosis is still not established (Bhatia et al., 2020; Maruno et al., 2022; Mutsaers and Nørregaard, 2022).

Accumulating evidence suggests that advanced glycation end products (AGEs) play an important role in the accumulation of ECM, which is consistently observed in diabetic and non-diabetic renal diseases associated with progressive glomerulosclerosis and tubulointerstitial fibrosis (Daroux et al., 2010). The receptor for AGEs (RAGE), which belongs to the immunoglobulin superfamily (Xie et al., 2008) is a multi-ligand transmembrane receptor with the capability to interact with several types of proteins, including AGEs, S100 proteins or calgranulins, the high-mobility group B1 (HMGB1, also known as “amphoterin”), and proteins with a *ß* fibrillary structure (Yan et al., 2008; Lin et al., 2009; Daroux et al., 2010). Binding of RAGE with these molecules activates a series of signal transduction pathways and transcription factors, including the mitogen-activated protein kinases (MAPK) and nuclear factor kappa B (NF-κB), leading to the production of reactive oxygen species and pro-inflammatory cytokines and consecutively inducing inflammation (Lin et al., 2009; Daroux et al., 2010). RAGE consists of an intracellular domain, a short transmembrane domain, and an extracellular ligand-binding domain (Xie et al., 2008). Soluble RAGE (sRAGE) is generated by the proteolytic cleavage of RAGE at the boundary between the extracellular and transmembrane portions or by alternative splicing of the RAGE gene (Santilli et al., 2009). Since sRAGE has the same ligand-binding specificity as that of RAGE, it may act as a decoy by competitively binding to proinflammatory ligands, preventing them from activating membrane-associated RAGE signaling (Xie et al., 2008; Lin et al., 2009; Santilli et al., 2009; Daroux et al., 2010). Blocking RAGE signaling using sRAGE has been revealed to be a potential therapeutic candidate for various diseases associated with inflammation, such as left ventricular hypertrophy, atherosclerosis, Alzheimer’s disease, and arthritis (Leonardis et al., 2012; Chuah et al., 2013; Lee et al., 2013; Lee et al., 2013; Farmer et al., 2014). Furthermore, the inflammatory process is known to play an important role in the pathogenesis and progression of tubulointerstitial nephritis (Boor et al., 2010; Kaissling et al., 2013; Y; Liu, 2011).

HMGB1 is a nuclear DNA-binding protein that resides inside the nucleus and can be released into the extracellular space by injured cells and macrophages (Lotze and Tracey, 2005; Bell et al., 2006). Once extracellular HMGB1 binds to its functional receptors, RAGE or toll-like receptors, a series of reactions can be triggered (Kokkola et al., 2005; Harris and Raucci, 2006; Yu et al., 2006; Tian et al., 2007; Chen et al., 2016). Recently, it was found that the RAGE-HMGB1 interaction is a potential signaling event in the development of renal tubulointerstitial fibrosis (Lynch et al., 2010; Rauvala and Rouhiainen, 2010; Chuang et al., 2013; Cheng et al., 2015). HMGB1 was also found to directly induce the accumulation of ECM in cultured renal tubular epithelial cells (Lynch et al., 2010; Cheng et al., 2015).

Based on these findings, it is surmised that the inhibition of RAGE signaling can provide a new therapeutic approach for the progression of renal fibrosis. However, the role of RAGE in renal tubulointerstitial fibrosis and the consequences of its inhibition by sRAGE are not fully understood.

Unilateral ureteral obstruction (UUO) is a well-established experimental tool for inducing tubulointerstitial fibrosis in obstructed kidneys (Furuta et al., 1993; Chevalier et al., 2009). Previous studies have shown that UUO leads to the release of damage-associated molecular patterns, including HMGB1, from tubular and interstitial cells in the kidney (Wyczanska and Lange-Sperandio, 2020). Thus, the interaction between RAGE and HMGB1 induced by UUO may serve as a crucial axis in the development of renal tubulointerstitial fibrosis and could potentially be targeted for therapeutic intervention.

Therefore, we investigated the therapeutic effects of sRAGE on HMGB1-induced renal tubular cell injury *in vitro* and UUO-induced renal tubulointerstitial fibrosis *in vivo*.

## 2 Materials and methods

### 2.1 *In vitro* studies: cell culture

The normal rat kidney proximal tubular epithelial cell line, NRK-52E, was purchased from the American Type Culture Collection (ATCC, Rockville, MD, United States) and was used for cell culture experiments. NRK-52E cells were cultured in Dulbeco’s Modified Eagle’s Medium (Invitrogen, Carlsbad, CA, United States) supplemented with 5% fetal bovine serum, 100 U/mL penicillin, 100 mg/mL streptomycin, and 26 mM sodium bicarbonate at 37°C in humidified 5% CO_2_ air. Subconfluent NRK-52E cells were serum-starved for 24 h, after which the media were replaced with serum-free medium containing HMGB1 (10 μg/mL) (A&RT, Daejeon, Korea), sRAGE (1 μg/mL) (A&RT, Daejeon, Korea), or HMGB1 with sRAGE. HMGB1 and sRAGE are recombinant human proteins. Twenty-four hours after the media change, the cells were harvested, and conditioned culture media were collected.

### 2.2 *In vivo* studies

All animal studies were conducted in accordance with the National Institute of Health Guide for the Care and Use of Laboratory Animals, and the institutional protocols were approved by the Committee for the Care and Use of Laboratory Animals at the Yonsei University College of Medicine, Seoul, Korea. Twenty-four Sprague-Dawley (SD) rats were grouped into four experimental groups with six rats each: sham operation with diluent, sham operation with sRAGE (4 μg/kg), UUO operation with diluent, and UUO operation with sRAGE. The animals were intraperitoneally treated with either the diluent or sRAGE 1 h before and every 48 h after the sham or UUO operation. The rats were sacrificed 10 days after the operation, and their kidneys were removed for histological evaluation and molecular biological analysis.

### 2.3 Generation of a renal tubulointerstitial fibrosis animal model by UUO

The UUO rat models were generated as previously described (Moriyama et al., 1998). Briefly, SD rats were anesthetized with sodium pentobarbital, after which a left flank incision was made. The left ureter was exposed, ligated with 6–0 silk sutures at two points, and cut between the two ligatures. Finally, the peritoneal membrane and the skin were sutured. The sham operation was performed as a control by following all steps of the UUO operation procedure, except for the ligation of the ureter.

### 2.4 Western blot analysis

Western blotting was performed as described previously (Oh et al., 2018). Primary antibodies to RAGE (Abcam, Cambridge, United Kingdom), HMGB1 (Cell Signaling, Inc., Beverly, MA, United States), fibronectin (DAKO, Glostrup, Denmark), type I collagen (Southern Biotech, Birmingham, AL, United States), α-smooth muscle actin (α-SMA), connective tissue growth factor (CTGF) (Abcam), intercellular adhesion molecule 1 (ICAM-1; R&D systems, Minneapolis, MN, United States), phospho-extracellular signal-regulated kinase (ERK)/ERK, phospho-p38/p38, phospho-Jun N-terminal kinase (JNK)/JNK, MyD88, phospho-NF-κB p65/NF-κB p65(Cell Signaling, Inc.,), or *ß*-actin (Sigma-Aldrich Corp., St. Louis, MO, United States) were obtained from commercial vendors. The membrane was washed once for 15 min and twice for 5 min in 1 × phosphate-buffered saline containing 0.1% Tween-20. The membranes were incubated in buffer A containing a 1:1000 dilution of horseradish peroxidase-linked goat anti-rabbit or anti-mouse immunoglobulin G (Santa Cruz Biotechnology, Inc., Santa Cruz, CA, United States). ImageJ software (NIH, Bethesda, MD, United States) was used to measure the band intensities and changes in the treated groups relative to the control cells or tissues. Especially for NF-κB protein expression, Western blotting was performed with nuclear and cytosolic fractions of cultured cells harvested from plates, which were separated using the NE-PER Nuclear and Cytoplasmic Extraction Reagents Kit (Thermoscientific, Waltham, MA, United States) according to the manufacturer’s protocol.

### 2.5 Enzyme-linked immunosorbent assay (ELISA)

Serum HMGB1 protein levels in SD rats were determined by ELISA using a rat ELISA kit (Novus Biologicals, United States, NBP3-06661), following the manufacturer’s instructions. Briefly, rat serum samples were placed in 96-well plates, incubated for 1 h at 37°C with biotin-conjugated antibodies targeting HMGB1, incubated with an enzymatic working solution for 30 min at 37°C, and incubated with a tetramethylbenzidine solution for 15 min at 37°C in darkness. Absorbance at 450 nm was measured to quantify protein abundance. Three biological replicates were used for ELISA analysis.

### 2.6 Immunohistochemistry and Masson’s trichrome staining

The removed kidney was sliced and fixed in 10% neutral-buffered formalin. The 5 μm-thick sections were processed for immunohistochemical (IHC) and Masson’s trichrome staining. For IHC staining, slides were deparaffinized, hydrated with ethyl alcohol, and washed with tap water. Antigen retrieval was performed using black-and-decker vegetable steamers in a 10 mM sodium citrate buffer for 20 min. Primary antibodies for RAGE, HMGB1, fibronectin, type I collagen, and ED-1 (Chemicon International, Inc., Billerica, MA, United States) were diluted to appropriate concentrations with 2% casein in bovine serum albumin and added to the slides, followed by overnight incubation at 4°C. After washing, a secondary antibody was added for 20 min, and the slides were then washed and incubated with a tertiary rabbit peroxidase-antiperoxidase complex (DAKO) for 20 min. Diaminobenzidine was added for 2 minutes, and the slides were counterstained with hematoxylin.

A semi-quantitative score for staining intensity was determined by examining at least 20 tubulointerstitial fields at × 400 magnification. Two investigators, in a blinded fashion, defined a positive staining intensity of 1+ to 4+ compared to a negative control (score = 0), in which IHC staining was performed without primary antibodies, based on the lightness and darkness of the brownish color using a digital image analyzer (MetaMorph version 4.6r5, universal Imaging, Downingtown, PA). The staining score was obtained by multiplying the intensity of staining by the percentage of tubulointerstitial staining for that intensity, and these numbers were added for each experimental animal to give the staining score [ = Σ (intensity of staining) × (% of tubulointerstitial staining with that intensity)]. To count the number of ED-1-positive cells, at least 20 fields of view of the tubulointerstitium per section at × 400 magnification were examined. ED-1 (also known as CD68 monoclonal antibody) was used as a marker for activated monocytes or macrophages.

### 2.7 Statistical analysis

All values are expressed as the mean ± standard error of the mean. Statistical analyses were performed using IBM SPSS Statistics for Windows version 20.0 (IBM Corp., Armonk, NY, United States). One-way analysis of variance was used to analyze the results, followed by Bonferroni’s post-hoc test or the Kruskal-Wallis nonparametric test for multiple comparisons, as appropriate. If significant differences were found using the Kruskal-Wallis test, they were further confirmed using the Mann-Whitney *U*-test. *p*-values less than 0.05 were considered statistically significant.

## 3 Results

### 3.1 *In vivo* results

#### 3.1.1 sRAGE abrogates UUO-induced tubulointerstitial fibrosis

**FIGURE 1:**
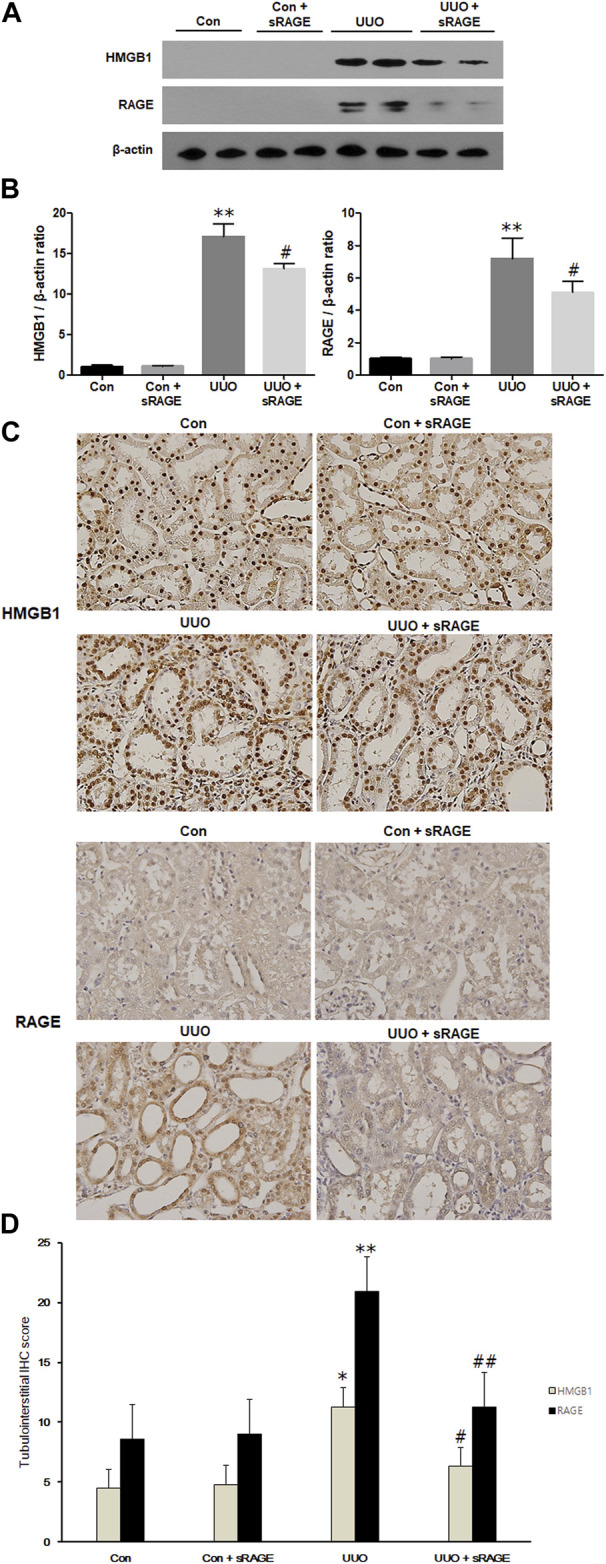
A representative Western blot (Representative of five blots) and immunohistochemical staining of HMGB1 and RAGE protein in the left kidney of sham-operated (Con), Con + sRAGE, UUO, and UUO + sRAGE rats. Compared to Con rats, there were 17.4- and 7.2-fold increases in HMGB1 and RAGE protein expression, respectively, in the left kidney of UUO rats treated with diluent (UUO), and administration of sRAGE significantly inhibited these increases in UUO rats **(A)**. Compared to Con and Con + sRAGE rats, tubulointerstitial HMGB1 **(B)** and RAGE **(C)** protein expression were significantly increased in UUO rats treated with diluent, and these increases were significantly abrogated by sRAGE treatment. The significant increases in IHC staining scores for HMGB1 and RAGE within the tubulointerstitium of UUO rats were significantly mitigated in the UUO rats treated with sRAGE **(D)**. *; *p* < 0.05 vs. Con and Con + sRAGE groups, **; *p* < 0.01 vs. Con and Con + sRAGE groups, #; *p* < 0.05 vs. UUO group, ##; *p* < 0.01 vs. UUO group. Each experiment **(A–C)** was repeated six times independently with similar results.

**FIGURE 2:**
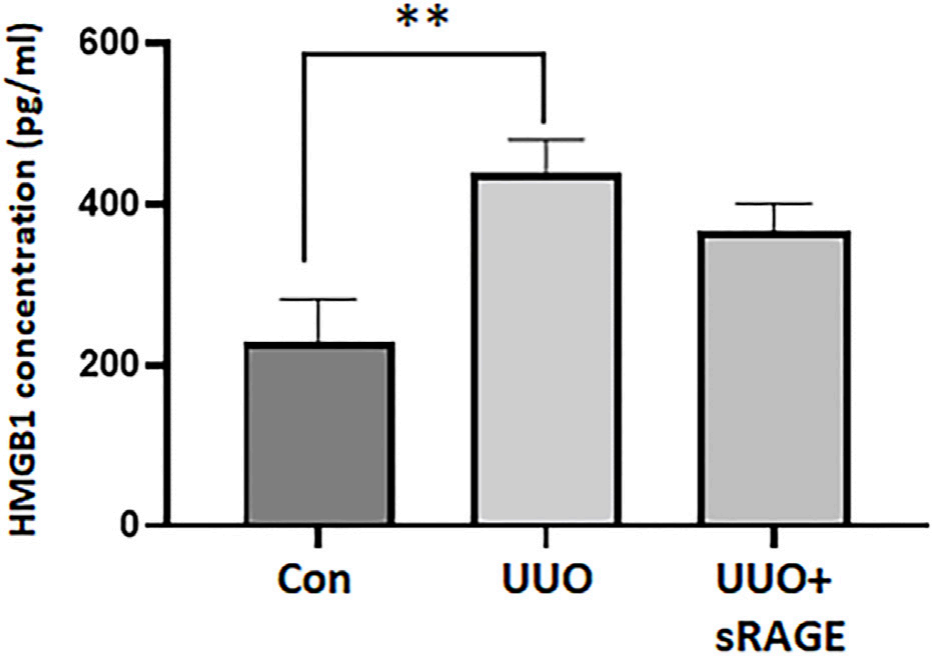
Serum HMGB1 levels measured by enzyme-linked immunosorbent assay of sham-operated (Con), UUO, and UUO + sRAGE rats. Compared to Con rats, serum concentration of HMGB1 was significantly higher in the UUO rats. sRAGE treatment decreased serum HMGB1 level slightly but it was not statistically significant. **; *p* < 0.01 vs. Con group.

We first evaluated HMGB1 and RAGE protein expression levels in the UUO rat models. Compared to sham-operated rats, renal HMGB1 and RAGE protein expression were significantly increased in the UUO rat models treated with diluent (*p* < 0.01). However, the administration of sRAGE significantly inhibited these increases in the UUO rat models (*p* < 0.05; Figure 1A). IHC staining for HMGB1 and RAGE proteins showed a pattern similar to the Western blot findings (*p* < 0.05 or 0.01; Figures 1B–D). Furthermore, we measured the serum HMGB1 levels in each group. Compared to sham-operated rats, serum HMGB1 levels were significantly higher in the UUO rat models (228.3 ± 53.4 pg/mL vs. 439.1 ± 41.3 pg/mL, *p* < 0.01), but sRAGE treatment in the UUO rat models resulted in a slight reduction in serum HMGB1 levels; however, it was not statistically significant (367 ± 33.7 pg/mL; *p =* 0.372; Figure 2).

**FIGURE 3:**
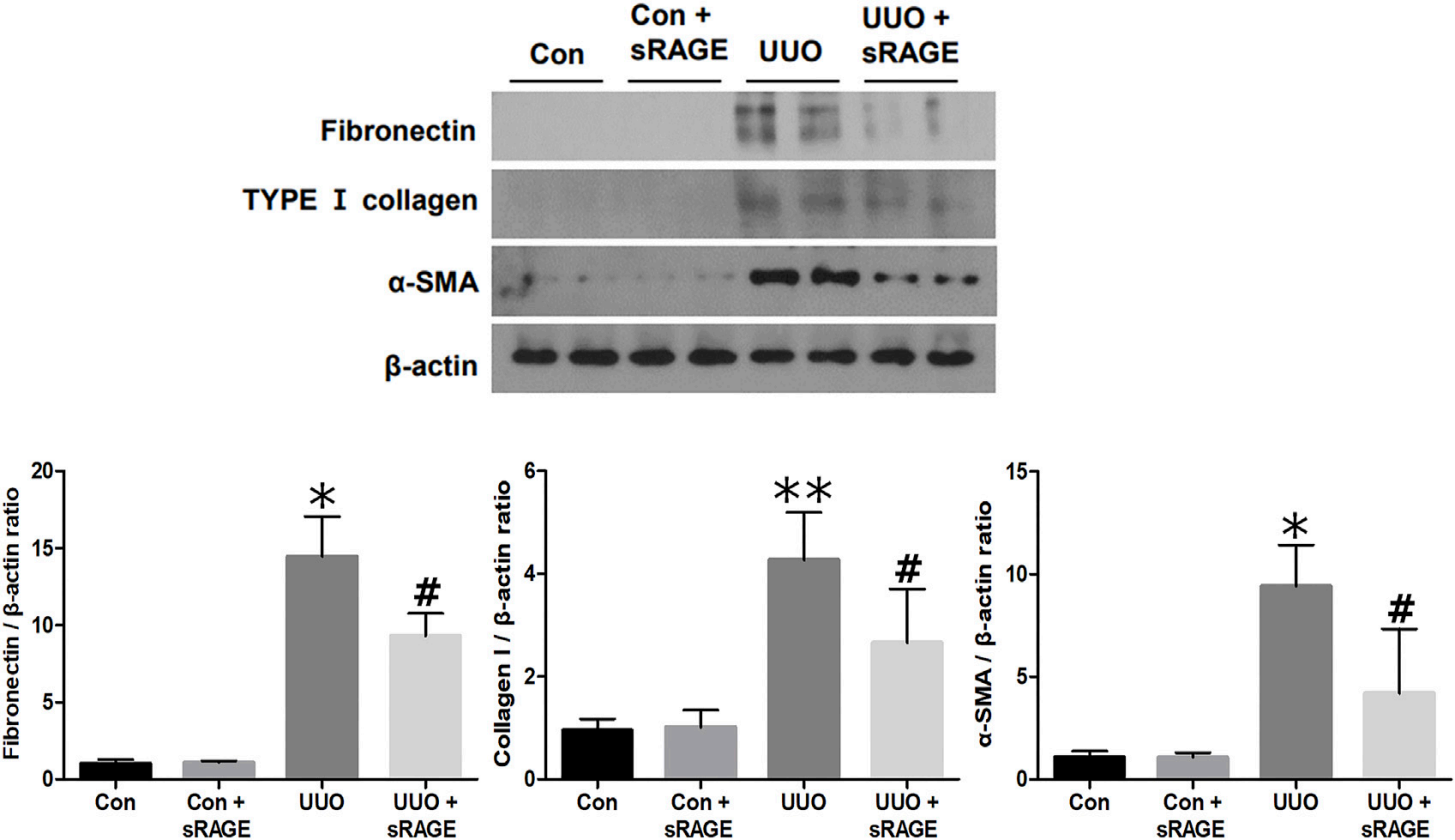
A representative Western blot of fibronectin, type I collagen, and α-SMA protein in the left kidney of sham-operated (Con), Con + sRAGE, UUO, and UUO + sRAGE rats (Representative of five blots). Compared to Con rats, there were 14.8-, 4.2-, and 9.4-fold increases in fibronectin, type I collagen, and α-SMA protein expression, respectively, in UUO rats, and these increases were significantly ameliorated by sRAGE treatment. *; *p* < 0.01 vs. Con and Con + sRAGE groups, **; *p* < 0.05 vs. Con and Con + sRAGE groups, #; *p* < 0.05 vs. UUO group. Each experiment was repeated six times independently with similar results.

**FIGURE 4:**
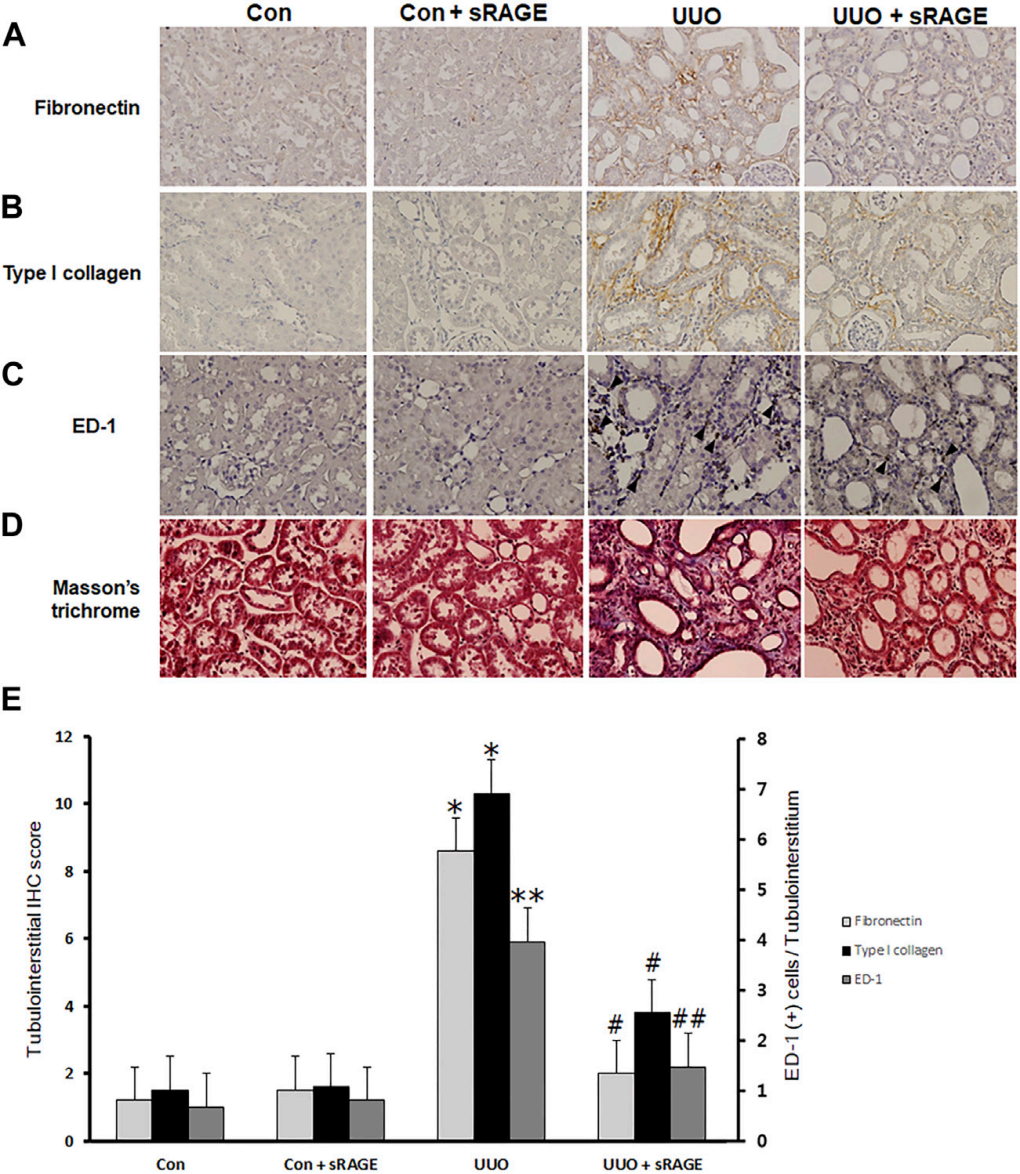
Immunohistochemical staining for fibronectin, type I collagen, and ED-1, and Masson’s trichrome staining with the left kidney tissues of sham-operated (Con), Con + sRAGE, UUO, and UUO + sRAGE rats. Compared to Con and Con + sRAGE rats, the protein expression of fibronectin **(A)** and type I collagen **(B)** within the tubulointerstitium were significantly increased in UUO rats, and sRAGE treatment significantly ameliorated these increases in UUO rats. **(C)** The significant increase in the number of ED-1-positive cells (arrowhead) within the tubulointerstitium of UUO rats was significantly inhibited by the administration of sRAGE. **(D)** Masson’s trichrome staining revealed that tubulointerstitial fibrosis was significantly severer in UUO rats relative to Con and Con + sRAGE rats, and this excessive fibrosis was significantly mitigated in sRAGE-treated UUO rats.*; *p* < 0.01 vs. Con and Con + sRAGE groups, **; *p* < 0.05 vs. Con and Con + sRAGE groups, #; *p* < 0.01 vs. UUO group, ##; *p* < 0.05 vs. UUO group. Each experiment **(A–D)** was repeated six times independently with similar results.

To examine the effect of sRAGE on UUO-induced tubulointerstitial fibrosis and macrophage accumulation, we analyzed the changes in fibronectin, type I collagen, α-SMA, and ED-1 protein expression using Western blot and IHC staining and assessed tubulointerstitial fibrosis by Masson’s trichrome staining. Renal fibronectin, type I collagen, and α-SMA protein expression were significantly increased in the UUO rat models compared to sham-operated rats (*p* < 0.05 or 0.01), but sRAGE treatment significantly ameliorated these increases (*p* < 0.05; Figure 3). Additionally, the UUO rat models showed increased IHC scores for fibronectin and type I collagen, which were alleviated by sRAGE treatment (*p* < 0.01; Figures 4A, B, E). Moreover, the number of infiltrating macrophages within the tubulointerstitium, as assessed by ED-1 staining, was significantly higher in the UUO group than in the control group, and sRAGE treatment significantly reduced the number of ED-1-positive cells in the UUO rat models (*p* < 0.05; Figure 4C). Furthermore, Masson’s trichrome staining revealed that tubulointerstitial fibrosis was significantly increased in the UUO rat models relative to control rats and was significantly mitigated in sRAGE-treated UUO rat models (Figure 4D).

#### 3.1.2 sRAGE attenuates the activation of the MAPK and NF-κB pathways in the UUO rat models

**FIGURE 5:**
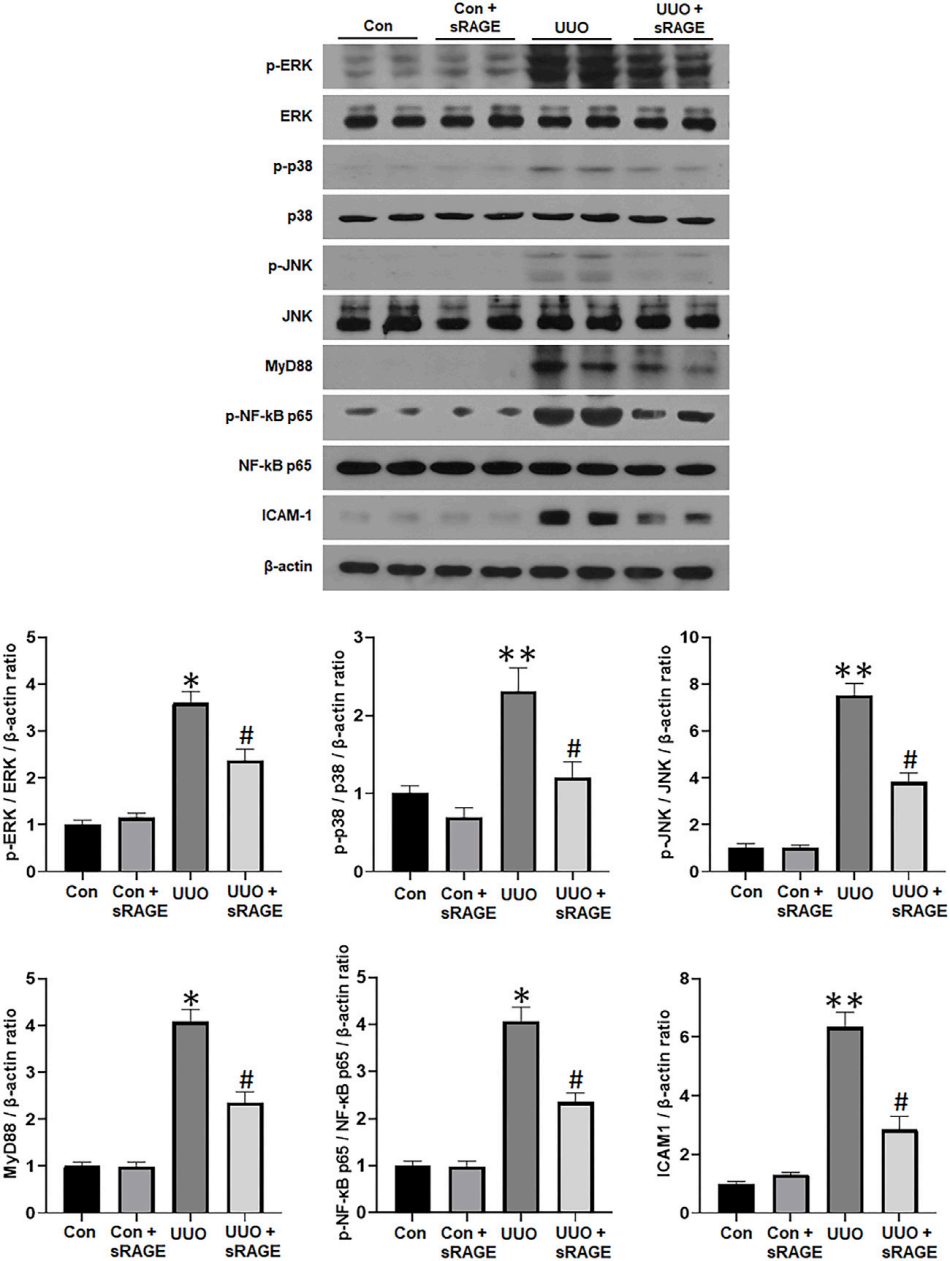
A representative Western blot of phospho-ERK/ERK, phospho-p38/p38, phospho-JNK/JNK, MyD88, phospho-NF-κB p65/NF-κB p65, and ICAM-1 protein in the left kidney of sham-operated (Con), Con + sRAGE, UUO, and UUO + sRAGE rats (Representative of five blots). Compared to Con rats, there were 6.3-, 2.3-, 7.5-, 4.1-, 4.0-, and 6.3-fold increases in phospho-ERK/ERK, phospho-p38/p38, phospho-JNK/JNK, MyD88, phospho-NF-κB p65/NF-κB p65, and ICAM-1 protein expression, respectively, in UUO rats, and these increases were significantly attenuated by sRAGE treatment. *; *p* < 0.01 vs. Con and Con + sRAGE groups, #; *p* < 0.05 vs. UUO group, **; *p* < 0.05 vs. Con and Con + sRAGE groups. Each experiment was repeated six times independently with similar results.

Finally, the effect of sRAGE on the components of the MAPK and NF-κB pathways was explored in the UUO rat models. As shown in Figure 5, phospho-ERK, phospho-p38, phospho-JNK, MyD88, phospho-NF-κB p65, and ICAM-1 protein expressions were significantly upregulated in the UUO rat models compared to sham-operated rats (*p* < 0.05 or 0.01), while sRAGE treatment in the UUO rat models significantly alleviated these changes (*p* < 0.05). These findings suggest that sRAGE can attenuate the activation of the MAPK and NF-κB pathways, leading to significant improvement in tubulointerstitial inflammation.

### 3.2 *In vitro* results

#### 3.2.1 sRAGE abrogates HMGB1-induced renal fibrosis in cultured NRK-52E cells

**FIGURE 6:**
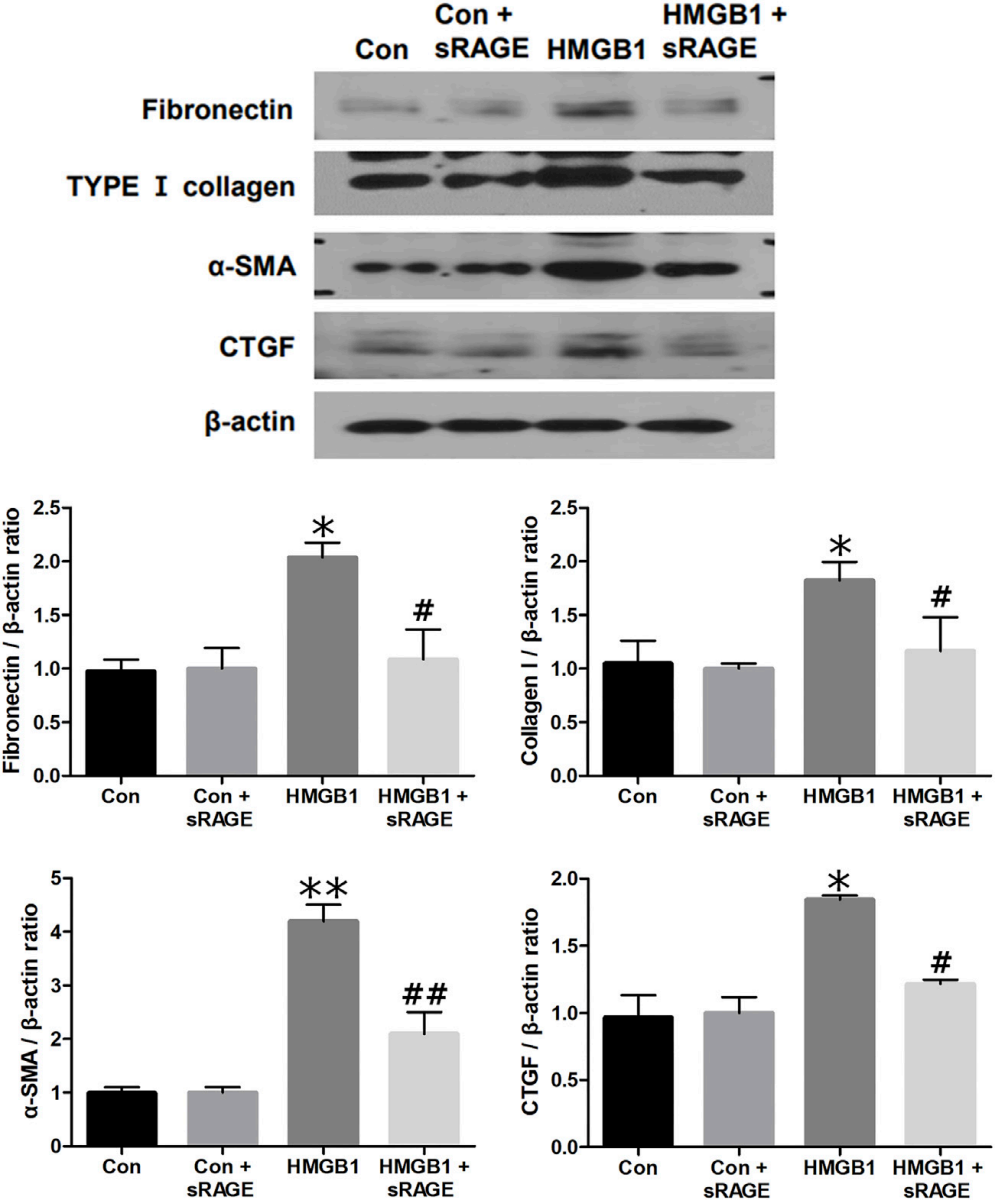
A representative Western blot of fibronectin, type I collagen, α-SMA, and CTGF protein in control (Con), Con + sRAGE, HMGB1 (10 μg/mL), and HMGB1+sRAGE (1 μg/mL) groups (Representative of six blots). Compared to Con cells, there were 2.1-, 1.9-, 4.2-, and 1.8-fold increases in fibronectin, type I collagen, α-SMA, and CTGF protein expression, respectively, in HMBG1-stimulated NRK-52E cells, and these changes were significantly abrogated by sRAGE treatment. *; *p* < 0.05 vs. Con and Con + sRAGE groups, #; *p* < 0.05 vs. HMGB1 group, **; *p* < 0.01 vs. Con and Con + sRAGE groups, ##; *p* < 0.01 vs. HMGB1 group. Each experiment was repeated six times independently with similar results.

**FIGURE 7:**
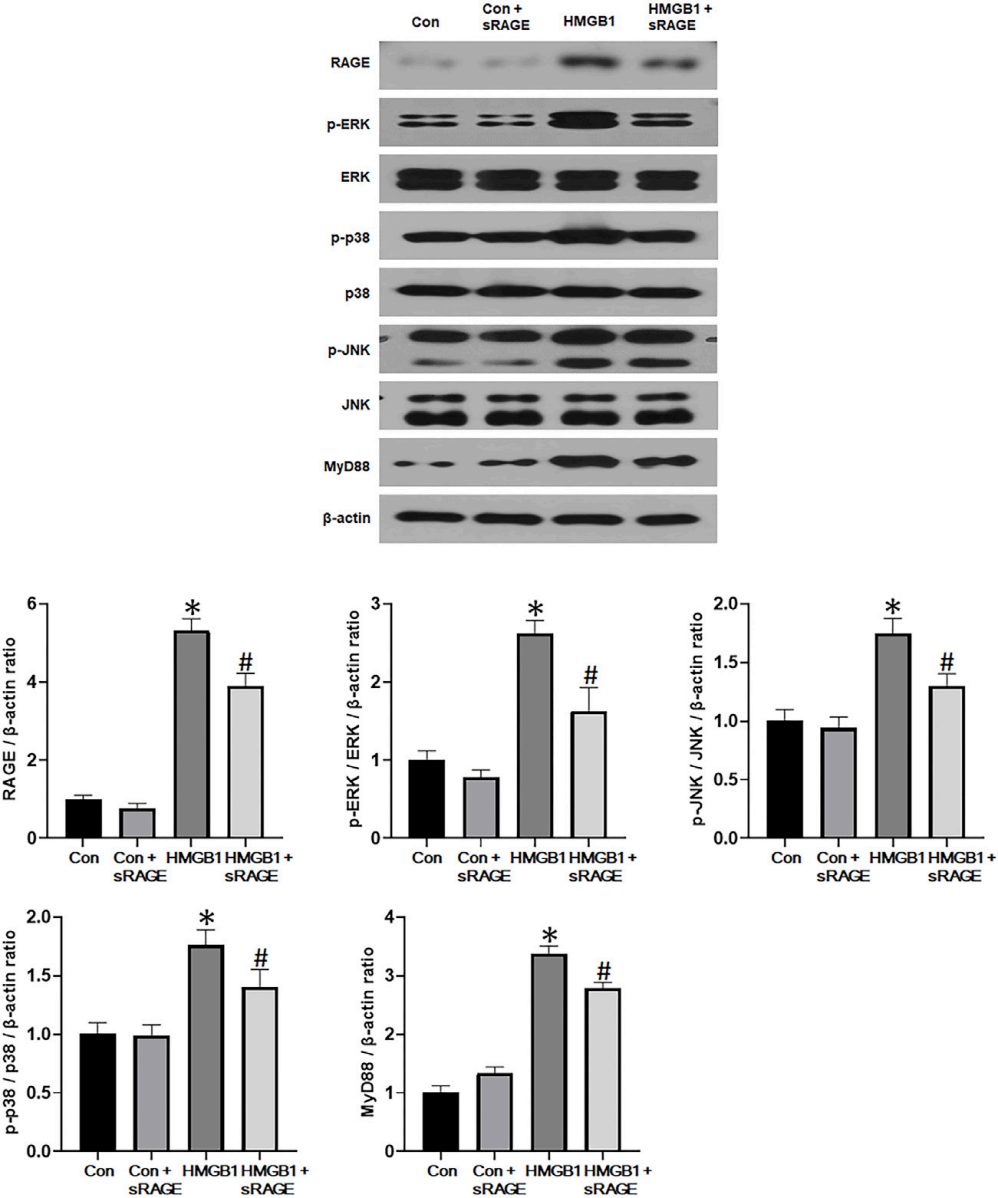
A representative Western blot of RAGE, phospho-ERK/ERK, phospho-p38/p38, phospho-JNK/JNK, and MyD88 protein in control (Con), Con + sRAGE, HMGB1 (10 μg/mL), and HMGB1+sRAGE (1 μg/mL) groups (Representative of six blots). Compared to Con cells, there were 5.3-, 2.6-, 1.8-, 1.8-, and 3.4-fold increases in RAGE, phospho-ERK/ERK, phospho-JNK/JNK, phospho-p38/p38, and MyD88 protein expression, respectively, in HMBG1-stimulated NRK-52E cells, and sRAGE significantly ameliorated these increases. *; *p* < 0.05 vs. Con and Con + sRAGE groups, #; *p* < 0.05 vs. HMGB1 group. Each experiment was repeated six times independently with similar results.

To investigate the potential of sRAGE in mitigating HMGB1-induced renal tubular epithelial cell injury, we assessed changes in fibronectin, type I collagen, α-SMA, and CTGF protein expressions in cultured NRK-52E cells. We found that HMGB1 treatment (10 μg/mL) significantly increased fibronectin, type I collagen, α-SMA, and CTGF protein expressions in cultured NRK-52E cells (*p* < 0.05 or 0.01). However, sRAGE treatment significantly abrogated this effect (*p* < 0.05 or 0.01; Figure 6). We also examined changes in RAGE, phospho-ERK/ERK, phospho-p38/p38, phospho-JNK/JNK, and MyD88 protein expression to elucidate the effect of sRAGE on HMGB1-activated MAPK in cultured NRK-52E cells. Our findings revealed that HMGB1 treatment significantly upregulated RAGE, phospho-ERK, phospho-p38, phospho-JNK, and MyD88 protein expressions in cultured NRK-52E cells compared to control cells (*p* < 0.05), while sRAGE treatment ameliorated HMGB-1 induced activation of MAPK and MyD88 pathways in cultured NRK-52E cells (*p* < 0.05; Figure 7).

#### 3.2.2 sRAGE attenuates activation of NF-κB in HMGB1-stimulated NRK-52E cells

**FIGURE 8:**
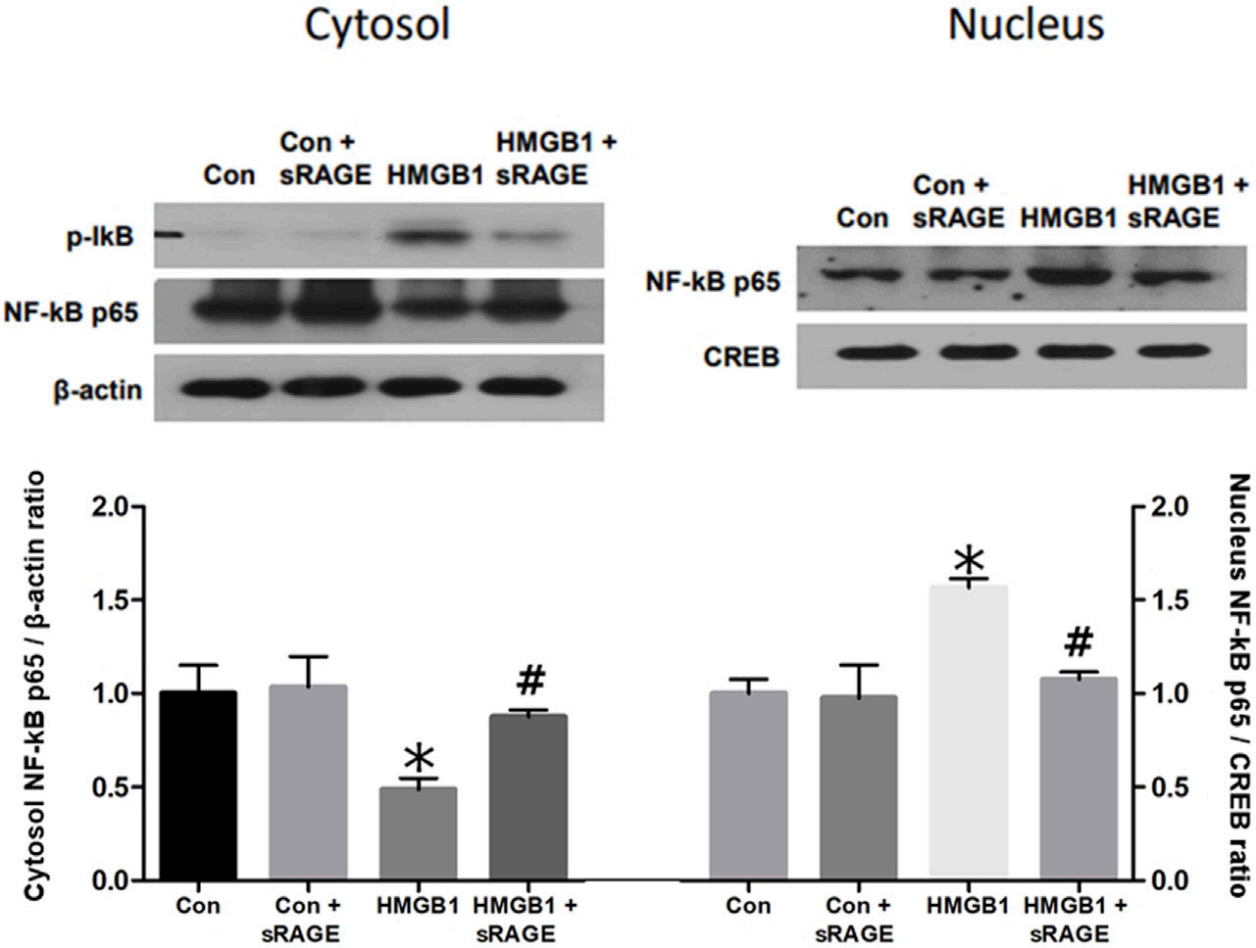
A representative Western blot of NF-κB p65 protein in control (Con), Con + sRAGE, HMGB1 (10 μg/mL), and HMGB1+sRAGE (1 μg/mL) groups (Representative of six blots). Compared to Con cells, there was a significant decrease in NF-κB p65 protein expression in the cytosolic fraction by 51.2% and a significant 1.6-fold increase in the nuclear fraction of HMBG1-stimulated NRK-52E cells, and these changes were significantly attenuated by sRAGE treatment. *; *p* < 0.05 vs. Con and Con + sRAGE groups, #; *p* < 0.05 vs. HMGB1 group. Each experiment was repeated six times independently with similar results.

Since HMGB1 was known to activate the NF-κB pathway, nuclear translocation of NF-κB was evaluated to determine the effects of sRAGE on HMGB1-induced activation of NF-κB by analyzing the changes in NF-κB p65 protein expression in both cytosolic and nuclear fractions. NF-κB p65 protein expression in the cytosolic fraction was significantly decreased 12 h after the administration of HMGB1 (*p* < 0.05), and, notably, sRAGE treatment dramatically restored the nuclear translocation of NF-κB p65 in HMGB-1 treated NRK-52E cells (*p* < 0.05; Figure 8).

## 4 Discussion

The present study demonstrated that RAGE expression was upregulated in HMGB1-treated NRK-52E cells and the kidneys of the UUO rat models, along with significantly increased fibrosis-related molecule expressions, such as fibronectin, type I collagen, and α-SMA. sRAGE treatment significantly abrogated these changes, indicating its potential as a therapeutic approach for various kidney diseases associated with renal fibrosis.

The study found that RAGE and HMGB1 expressions in the kidneys of the UUO rat models significantly increased, along with increased fibrosis-related protein expression and tubulointerstitial fibrosis. RAGE protein expression levels and these fibrosis-related molecules were also significantly increased in HMBG1-stimulated NRK-52E cells. These findings suggest that tubular cell injury induced by UUO might lead to an increase in HMGB1 expression and that increased HMGB1 in the kidneys of the UUO rat models and exogenous HMGB1 in cultured tubular cells could bind to RAGE. This RAGE-HMGB1 interaction could further induce RAGE expression through a positive feedback loop. Consistent with our study, previous studies have shown that the binding of various ligands, including HMGB1, to RAGE results in the upregulation of RAGE. The effects of RAGE and its ligands can be amplified in various diseases, such as vascular disease, diabetes, cancer, hepatic fibrosis, chronic airway disease, and neurodegenerative disease (Han et al., 2011; Zhao et al., 2014; Xia et al., 2015; Chen et al., 2016).

RAGE is a multiligand transmembrane receptor that interacts with various proteins, including AGEs (Xie et al., 2008; Kalea et al., 2009; Lin et al., 2009). It is primarily expressed in vascular smooth muscle and endothelial cells, mononuclear phagocytes, neurons, and lung alveolar epithelial cells (Kalea et al., 2009; Lin et al., 2009; Daroux et al., 2010). In the kidney, RAGE has been identified on the surfaces of glomerular endothelial cells, podocytes, mesangial cells, and tubular epithelial cells (Kalea et al., 2009; Lin et al., 2009; Daroux et al., 2010). When RAGE interacts with its ligands, it activates intracellular signaling pathways, such as the Janus kinase/signal transducer and activator of transcription, nuclear factor of activated T-cells 1, PI3K/AKT/GSK3β/β-catenin, and MAPK/NF-κB pathways (Huang et al., 2001; Han et al., 2011; Chen et al., 2016). The resulting cellular responses can include several types of events, like pro-inflammatory, pro-fibrotic, pro-coagulant, angiogenic, epithelial-mesenchymal transition, or even repair processes, depending on the ligand, environment, and developmental stage (Daroux et al., 2010; Chuah et al., 2013; Chen et al., 2016). Previous studies correlated the engagement of RAGE with various ligands with the pathogenesis of several fibrotic diseases in different organs, including hepatic fibrosis, idiopathic pulmonary fibrosis, myocardial infarction, and diabetic cardiomyopathy (Ku et al., 2015; Kyung et al., 2014; T. W; Lee et al., 2014; Xia et al., 2015; Yamagishi and Matsui, 2015; Zhao et al., 2014). Blocking the RAGE-ligand axis has protected these organs against pathological fibrosis (Ku et al., 2015; T. W; Lee et al., 2014; Xia et al., 2015; Yamagishi and Matsui, 2015). A recent study by Liu et al. demonstrated that renal tubulointerstitial fibrosis in UUO model mice is mediated by RAGE in proximal tubular cells through the upregulation of autophagy (B. Liu et al., 2022), partially supporting our findings. However, another recent study showed that the interaction between HMGB1 and RAGE induces epithelial-mesenchymal transition in human airway epithelial cells (Furuta et al., 1993). Additionally, UUO-induced renal fibrosis is associated with epithelial-mesenchymal transition steps mediated by S100A8/A9 (Tammaro et al., 2018). These studies suggest that the mechanism of epithelial-mesenchymal transition may partially contribute to renal tubulointerstitial fibrosis, which we were unable to address in our study.

As sRAGE binds to AGEs and acts as a decoy, it can competitively inhibit the activation of RAGE (Kalea et al., 2009; Lin et al., 2009; Han et al., 2011). Previous studies have demonstrated the beneficial effects of sRAGE in various experimental disease models. For instance, intraperitoneal administration of sRAGE attenuated albuminuria, glomerulosclerosis, and glomerular basement membrane thickening in db/db mice (Wendt et al., 2003). Additionally, sRAGE treatment significantly abrogated proteinuria and histological renal damage and reduced inflammation in lupus-prone mice (S. W. Lee et al., 2013). In autosomal dominant polycystic kidney disease, sRAGE inhibits the progression of the disease by down-regulating cell proliferation (E. J. Lee et al., 2015). Previous studies have identified the therapeutic effects of sRAGE in acute disease models associated with inflammation. However, the effect of sRAGE in chronic disease models, such as renal tubulointerstitial fibrosis, has not been extensively explored. In the present study, we found, for the first time, that sRAGE could significantly ameliorate the increased fibrosis-related molecule protein expressions in the kidneys of the UUO rat models and HMGB1-stimulated renal tubular cells. These findings indicate that sRAGE can be used to prevent and retard renal tubulointerstitial fibrosis in CKD. Recently, a DNA aptamer directed against RAGE (RAGE-aptamer) has shown potential as a novel therapeutic tool in streptozocin-induced diabetic rats and mice with deoxycorticosterone acetate or salt-induced renal injury (Matsui et al., 2017; Taguchi et al., 2018). Additionally, the RAGE-specific inhibitor FPS-ZM1 has alleviated renal injury in spontaneously hypertensive rats (Y. Liu et al., 2020). Taken together, our findings suggest that blocking the activation of RAGE using a specific antagonist could be a viable therapeutic approach.

Furthermore, we observed elevated serum HMGB1 levels in the UUO rat models. A recent study has demonstrated that sRAGE can attenuate the increase in HMGB1 levels mediated by angiotensin II stimulation *in vitro* (Lim et al., 2018). However, the present study did not show a significant reduction in serum HMGB1 levels with sRAGE treatment. This suggests that sRAGE might not only attenuate serum HMGB1 but also downstream signaling pathways through HMGB1 in the UUO-induced fibrosis model.

RAGE signaling activates MyD88 and MAPK, promoting cytokine production and activating cell signaling pathways involved in fibrosis and apoptosis (Han et al., 2011; Sakaguchi et al., 2011; Wang et al., 2013). The results of the present study demonstrated that sRAGE treatment significantly attenuated MyD88 protein expression and phosphorylation of ERK, p38, and JNK in the kidneys of the UUO rat models and HMGB1-stimulated NRK-52E cells. Another important downstream signaling pathway through RAGE is the NF-κB signaling pathway, which is known to lead to renal damage by inducing the transcription of inflammation-associated genes (Chuang et al., 2013), including ICAM-1, a cell surface glycoprotein that plays a major role in the infiltration of macrophages and monocytes (Frommhold et al., 2010). Infiltrated and activated macrophages or monocytes release lysosomal enzymes, nitric oxide, reactive oxygen species, tumor necrosis factor-α, interleukin-1, and TGF-α, resulting in renal injury (Furuta et al., 1993; Tesch et al., 1999). Previous studies have suggested that RAGE is involved in the inflammation process by mediating leukocyte recruitment (Frommhold et al., 2010; Chuah et al., 2013). Our study demonstrated that sRAGE treatment reduced phospho-NF-κB and ICAM-1 expression in the kidneys of the UUO rat models. Taken together with the changes in MAPK, NF-κB, and ICAM-1 molecules, it can be inferred that the beneficial effect of sRAGE on renal fibrosis was partly attributed to its inhibitory effect on these MAPK and subsequent inflammatory pathways.

In summary, this study showed for the first time that sRAGE can abrogate renal tubulointerstitial fibrosis both *in vitro* and *in vivo* by inhibiting the activation of MAPKs and NF-κB, suggesting that RAGE might be an important mediator in the pathogenesis of renal tubulointerstitial fibrosis. Therefore, sRAGE represents a potential therapeutic candidate for various kidney diseases associated with renal tubulointerstitial fibrosis.

## Data Availability

The raw data supporting the conclusion of this article will be made available by the authors, without undue reservation.
